# Recombinant Fasciola hepatica Fatty Acid Binding Protein as a Novel Anti-Inflammatory Biotherapeutic Drug in an Acute Gram-Negative Nonhuman Primate Sepsis Model

**DOI:** 10.1128/Spectrum.01910-21

**Published:** 2021-12-22

**Authors:** Jose J. Rosado-Franco, Albersy Armina-Rodriguez, Nicole Marzan-Rivera, Armando G. Burgos, Natalie Spiliopoulos, Stephanie M. Dorta-Estremera, Loyda B. Mendez, A. M. Espino

**Affiliations:** a Department of Microbiology and Medical Zoology, University of Puerto Rico, Medical Sciences Campus, San Juan, Puerto Rico; b Caribbean Primate Research Center University of Puerto Rico, Medical Sciences Campus, Toa Baja, Puerto Rico; c Department of Science & Technology, Universidad Ana G. Mendez, Recinto de Carolina, Carolina, Puerto Rico; Emory University School of Medicine

**Keywords:** *Fasciola hepatica*, fatty acid binding protein, sepsis, rhesus macaques, cytokines

## Abstract

Due to their phylogenetic proximity to humans, nonhuman primates (NHPs) are considered an adequate choice for a basic and preclinical model of sepsis. Gram-negative bacteria are the primary causative of sepsis. During infection, bacteria continuously release the potent toxin lipopolysaccharide (LPS) into the bloodstream, which triggers an uncontrolled systemic inflammatory response leading to death. Our previous research has demonstrated *in vitro* and *in vivo* using a mouse model of septic shock that Fh15, a recombinant variant of the Fasciola hepatica fatty acid binding protein, acts as an antagonist of Toll-like receptor 4 (TLR4) suppressing the LPS-induced proinflammatory cytokine storm. The present communication is a proof-of concept study aimed to demonstrate that a low-dose of Fh15 suppresses the cytokine storm and other inflammatory markers during the early phase of sepsis induced in rhesus macaques by intravenous (i.v.) infusion with lethal doses of live Escherichia coli. Fh15 was administered as an isotonic infusion 30 min prior to the bacterial infusion. Among the novel findings reported in this communication, Fh15 (i) significantly prevented bacteremia, suppressed LPS levels in plasma, and the production of C-reactive protein and procalcitonin, which are key signatures of inflammation and bacterial infection, respectively; (ii) reduced the production of proinflammatory cytokines; and (iii) increased innate immune cell populations in blood, which suggests a role in promoting a prolonged steady state in rhesus macaques even in the presence of inflammatory stimuli. This report is the first to demonstrate that a *F. hepatica*-derived molecule possesses potential as an anti-inflammatory drug against sepsis in an NHP model.

**IMPORTANCE** Sepsis caused by Gram-negative bacteria affects 1.7 million adults annually in the United States and is one of the most important causes of death at intensive care units. Although the effective use of antibiotics has resulted in improved prognosis of sepsis, the pathological and deathly effects have been attributed to the persistent inflammatory cascade. There is a present need to develop anti-inflammatory agents that can suppress or neutralize the inflammatory responses and prevent the lethal consequences of sepsis. We demonstrated here that a small molecule of 14.5 kDa can suppress the bacteremia, endotoxemia, and many other inflammatory markers in an acute Gram-negative sepsis rhesus macaque model. These results reinforce the notion that Fh15 constitutes an excellent candidate for drug development against sepsis.

## INTRODUCTION

As part of their immunomodulatory mechanisms, helminths establish a regulatory anti-inflammatory immune response in their mammalian host with a prominent T helper-2/T regulatory (Th2/Treg) immune profile (1, 2), which is thought to be mutually beneficial for the host and parasite because it protects the host from severe consequences of inflammatory responses while preventing the elimination of worms from the host (3). Thus, a large number of human and animal studies have demonstrated that helminth infections could be used to ameliorate or prevent inflammatory diseases (4–8). In fact, severe sepsis is significantly less frequent in persons carrying chronic helminth infections than in nonparasitized persons (1). These studies have helped to incorporate helminth infections into the expanded “hygiene hypothesis” (9). Fasciola hepatica, one of the most prevalent parasitic Platyhelminthes (10–12), is not an exception. However, because of the pathogenicity and negative impact that *F. hepatica* exerts on the health of animals and humans, infection with this parasite cannot be used to treat inflammatory diseases in humans. Additionally, the immune regulation associated with *F. hepatica* infection lacks specificity and results in a compromised immune system unable to respond effectively to bystander infections (13, 14). We consider it more judicious to identify and purify defined immune-modulatory molecules produced by the parasite, which have the potential for drug development, and to characterize their precise mechanism of action. Owing to its extraordinary capabilities to modulate the host immune system, *F. hepatica* constitutes an enormous “pharmacopeia.” As soon as the parasite invades the gut wall, it initiates a complex interaction with various host immune cells (e.g., macrophages or dendritic cells). The parasite secretes a myriad of immunomodulatory molecules termed excretory-secretory products (ESPs) that direct the host immune response toward a nonprotective Th2/Treg environment with suppressed Th1 immunity, which allows the parasite to persist in the host for a long period of time (15–18). Proteomic studies demonstrate that one of these molecules belong to the fatty acid binding protein family (FABP). Fasciola hepatica FABPs are highly abundant either in the surface or in the internal compartments of the adult parasite (19, 20). Fasciola hepatica FABPs are known antioxidant molecules (21) that have been used extensively as vaccine candidates against fascioliasis or schistosomiasis (22–24). In a previous study, we demonstrated that a single therapeutic dose of recombinant *F*. *hepatica* FABP (Fh15; 50 μg) given to mice 1 h after exposure to a lethal lipopolysaccharide (LPS) dose challenge significantly suppressed the cytokine storm by concurrently modulating the dynamics of macrophages in the peritoneal cavity and the activation status of spleen macrophages in a mouse model of septic shock (25).

Since nonhuman primates (NHPs) and humans originated from a common phylogenetic ancestor, they share similar physiological and anatomical features and display a similar cytokine response to intravenous (i.v.) endotoxin and live bacteria (26). Moreover, their large size allows for invasive monitoring, tissue collection, and serial phlebotomy (27). Thus, NHPs represent a more relevant preclinical model than the rodent model to study the inflammatory responses during the acute phase of sepsis. With these advantages in mind, in a previous study, we developed a rhesus macaque model of septic shock induced by intravenous administration of live Escherichia coli to identify inflammation-associated markers during the early phase of sepsis in rhesus macaques (28). As a result of that study, we were able to determine that bacteremia was present in all animals from 30 minutes to 4 hours following E. coli infusion, whereas endotoxin, C-reactive protein (CRP), and Procalcitonin (PCT) were detected during the full time course suggesting an ongoing inflammatory process caused by an active bacterial infection. Similarly, tumor necrosis factor alpha (TNF-α) was detected at 2 h, whereas interleukin-6 (IL‐6), IL‐12, and interferon gamma (IFN-γ) were detected after 4 h of E. coli infusion (28). The present study is a proof of concept to assess whether these inflammatory markers can be suppressed when a single low dose of Fh15 is administered i.v. as an isotonic infusion 30 min before a challenge with a live E. coli infusion. Knowing that Fh15 is a protein molecule and its development as a drug could be hampered due to limited half-life and immunogenicity (capacity to induce the formation of anti-Fh15 antibodies), the present study also aimed to determine the duration of Fh15 in circulation as well as to determine whether its immunogenicity could hamper the anti-inflammatory effect.

The present study is the first to demonstrate that although the half-life of Fh15 in circulation is short, it is able to significantly suppress bacteremia, endotoxemia, CRP, and PCT during the early phase of an E. coli-induced acute sepsis (8 hours) in a rhesus macaque model. Although the reductions in the proinflammatory cytokine/chemokine were not found significant, all monkeys that received the Fh15 treatment showed a trend of reduced concentrations in plasma of all proinflammatory cytokines and chemokines measured, while concurrently modulating the population of innate immune cells in the blood.

## RESULTS

### Physiological parameters before and after sepsis induction.

At baseline, body temperature (BT), heart rate (HR), and respiratory rate (RR) of all rhesus macaques involved in the study were at normal values ranging between 33.4 and 37.2°C (median, 35.9°C), 85 to 160 beats/min (median, 114 beats/min), and 13 to 25 breaths/min (median, 21 breaths/min), respectively. The mean arterial blood pressure (MAP) ranged between 33 and 78 mm Hg (median, 51 mm Hg). These MAP measurements were lower than expected. According to recent literature, oscillometric monitoring of BP is often underestimated (29, 30). None of the monkeys in the experiment died because of the experimental treatment. Monkeys that received the E. coli infection were euthanized at 8 h of experimentation. At the conclusion of the experiment, the physiological parameters were similar or slightly higher than the baseline for most of animals (see Table S2 in the supplemental material). For experimental reasons explained in the Materials and Methods section, monkeys in the Fh15 group were allowed to recover after 8 h. Therefore, it was not possible to determine by postmortem examination whether the Fh15 dose administered induced any gross type of toxicity. However, the physiological parameters of these animals remained at normal values during the entire time course of the experiment, and after recovering and returning to their respective cages, they remained healthy for 3 months (Table S2). When monkey MA035 received the second Fh15 treatment followed by the E. coli infection (group Fh15-Fh15-E. coli), its MAP decreased from 41 to 21 mm Hg and the RR dropped from 20 to 5 breaths per minute (bpm). Despite this abrupt decrease of RR, the monkey did not die prematurely. Postmortem examination of monkeys revealed no gross abnormalities with an exception of splenomegaly, which was noticed in MA035, and lymphadenopathy that was observed in all monkeys from the E. coli group.

### Development of antibodies to Fh15 and dynamics of Fh15 antigenemia.

**FIG 1:**
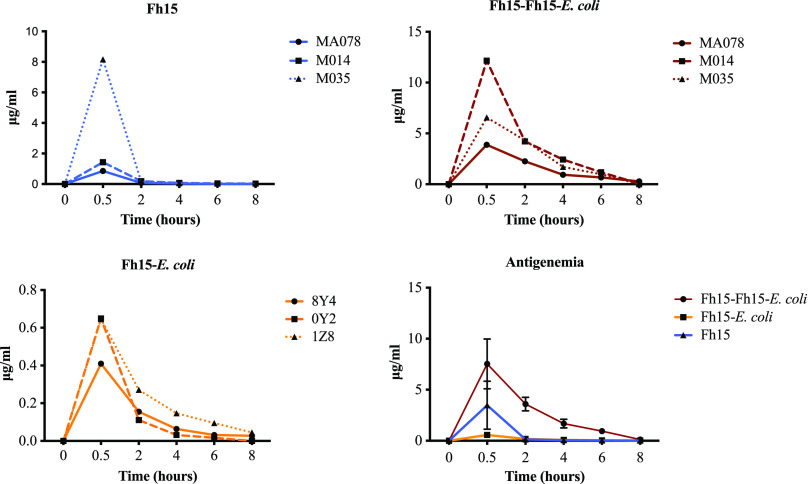
Levels of antigenemia in plasma samples from rhesus macaques treated with Fh15. A homemade sandwich ELISA was optimized to detect circulating Fh15 in plasma samples. The assay uses a rabbit anti-Fh15 IgG antibody as the capturing antibody and a rabbit anti-Fh15-IgG conjugate with HRP as the detecting antibody. Plasma samples were collected from rhesus macaques at different time points, as follows: 0 h (baseline), 30 min, 2 h, 4 h, 6 h, and 8 h during the experiment. The group termed Fh15 comprised monkeys (*n* = 3) that received only the i.v. infusion with 15 mg Fh15. The group termed Fh15-E. coli comprised animals (*n* = 3) that received the i.v. infusion containing 12 mg Fh15 followed by the E. coli infection (live E. coli 10^10^ CFU/kg body wt.). The group termed Fh15-Fh15-E. coli comprised animals from the Fh15 group that 3 months later received a second i.v. infusion with 12 mg Fh15 followed by the E. coli infection.

A double-antibody sandwich enzyme-linked immunosorbent assay (ELISA) optimized as described in the Materials and Methods was used to measure levels of circulating Fh15 in the plasma of Fh15-infused rhesus macaques. Antibodies against Fh15 were measured by a previously optimized indirect ELISA (31). As expected, none of the rhesus macaques in the Fh15 or Fh15-E. coli groups had antibodies or circulating Fh15 in the plasma samples collected at baseline, which confirmed that none of animals had been previously exposed to this antigen or were infected with parasites that potentially could induce cross-reactive antibodies to Fh15. When the plasma samples collected from animals in the Fh15 group (monkeys MA014, MA035, and MA078) were tested for antigenemia, we found maximal Fh15 concentrations (3.48 ± 3.31 μg/mL) at 30 min following the infusion, which declined at 2 h to an average of 0.135 ± 0.048 μg/mL and were undetectable at subsequent time points. The monkey MA035 was the main contributor to the antigenemia of this group, with an average concentration of 8.16 ± 0.3 μg/mL at 30 min. In the monkeys from Fh15-E. coli group (8Y4, 0Y2, and 1Z8), the average Fh15 concentration at 30 min was 6.12-fold lower than that in the Fh15 group (mean, 0.568 ± 0.11 μg/mL). At 2 h, the antigenemia declined in this group to an average of 0.179 ± 0.067 μg/mL and was undetectable thereafter. As expected, the plasma sample collected prior to the second Fh15 infusion administered to animals from the Fh15-Fh15-E. coli group (MA014, MA035, and MA078) tested positive for antibodies, with anti-Fh15 antibody titers among ∼1:200 to 1:400. The average plasma Fh15 concentration in these animals at 30 min was 7.53 ± 3.44 μg/mL, which is 2.16-fold higher than that observed in the Fh15 group and 13.25-fold higher than that observed in the Fh15-E. coli group. Although the monkey MA014 was the main contributor to the antigenemia at 30 min (12 μg/mL), the other two monkeys (MA078 and MA035) also showed high Fh15 concentrations (3.88 and 6.56 μg/mL, respectively) (Fig. 1). At 2 h, the antigenemia of this group decreased to an average of 3.599 ± 0.95 μg/mL; at 4 h, it was 1.685 ± 0.612 μg/mL, and at 6 h, it still was detectable with concentrations of 0.94 ± 0.21 μg/mL. After 8 h, circulating Fh15 was no longer detected.

### Fh15 suppresses the bacteremia, endotoxemia, and production of acute-phase proteins, which are sepsis hallmarks.

**FIG 2:**
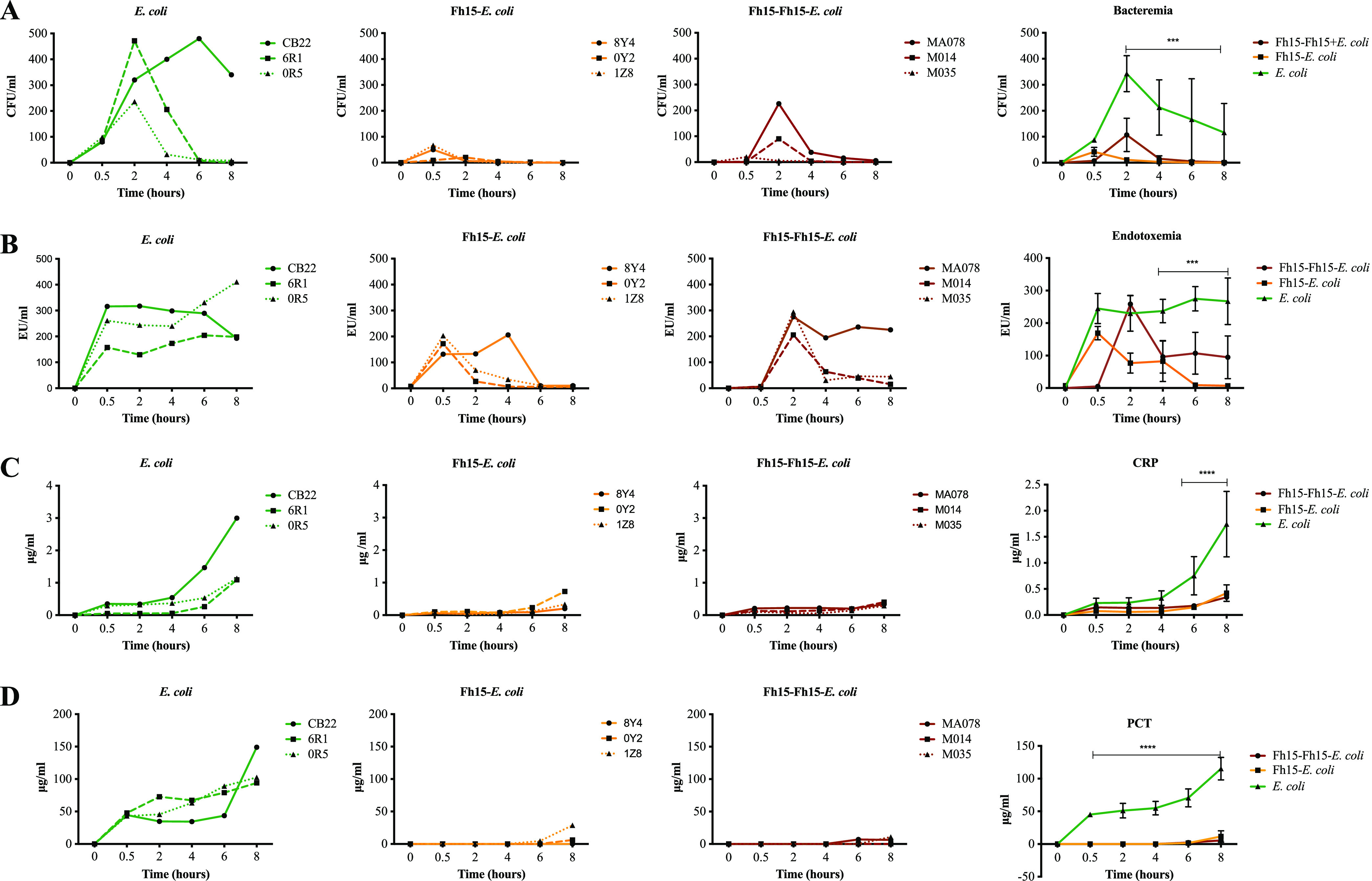
Fh15 decreases bacteremia, endotoxemia, C-reactive protein, and procalcitonin in rhesus macaques during an acute lethal sepsis. Rhesus macaques were allotted into three experimental groups (*n* = 3) each. The group termed E. coli is the control group comprised by monkeys that received only a lethal i.v. infusion with live E. coli 10^10^ CFU/kg of body wt. The group termed Fh15-E. coli comprised monkeys (*n* = 3) that received the i.v. infusion containing 12 mg Fh15 followed by the E. coli infection. The group termed Fh15-Fh15-E. coli comprised animals from a group that had received a primary i.v. infusion with 12 mg Fh15. Three months later, these monkeys received a second 12-mg Fh15 treatment followed by the E. coli infection. (A) The graphs represent the levels of viable bacteria for each single monkey of each experimental group as well as the comparison among the significant reductions in bacteremia showed by Fh15-E. coli and Fh15-Fh15-E. coli compared with the E. coli control group (***, *P* = 0.0001). Represents the levels of plasma endotoxin (LPS) (***, *P* < 0.0001) (B), C-reactive protein (***, *P* = 0.004) (C), and procalcitonin (****, *P < *0.0001) (D). Statistical significance between control group and experimental groups was determining by ANOVA or Student’s *t* test using GraphPad Prism 8. For all tests, a *P* value of <0.05 was considered significant.

In agreement with our previous observations using the rhesus macaques model of sepsis (28), monkeys that received only the bacterial infusion (CB22, 6R1, and 0R5) had the highest levels of bacteremia, which were high from 30 min following the bacterial infusion administration (87.33 ± 7.71 CFU/mL). Overall, the bacteremia in this group increased progressively until reaching a peak at 2 h (342.66 ± 97.67 CFU/mL). At 4 h, the average bacteremia in this group was 212.66 ± 150.30 CFU/mL. One monkey of this group (CB22) had high and persistent levels of bacteremia at every time point (average, 385 ± 62.24 CFU/mL), whereas in the other two animals (6R1 and 0R5), the average number of viable bacteria declined dramatically to 10 ± 2 CFU/mL and 4 CFU/mL at 6 h and 8 h, respectively (Fig. 2A).

As expected, the levels of plasma LPS in all animals of the E. coli group increased abruptly at 30 min (244.81 ± 65.76 endotoxin unit [EU]/mL) and remained at very high levels throughout the experiment (252.3 ± 76.8 EU/mL) (Fig. 2B). The increase of plasma endotoxin is a logical consequence of the bacteria disruption and consequent releasing of LPS to the bloodstream, an observation that also should be seem in our previous study developing the rhesus model of endotoxemia (28). The C-reactive protein (CRP) (Fig. 2C), an acute phase protein that typically increases in plasma during the inflammatory process (32), was detectable at 6 h and reached maximal values at 8 h following the E. coli infusion (1.976 ± 1.218 μg/mL). Procalcitonin (PCT) (Fig. 2D), a marker of bacterial infection and a predictor of sepsis in humans (33), was detected from 30 min and increased sequentially until reaching maximal levels at 8 h following E. coli infusion (115.21 ± 24.23 pg/mL). The animals that received only the Fh15 infusion did not develop bacteremia, endotoxemia, CRP, or PCT at any time throughout the study (data not shown). This finding is clear evidence that the Fh15 infusion was prepared in a pyrogenic-free manner.

An important finding of the present study is that the number of viable bacteria in the Fh15-E. coli group was drastically lower at every time point than that of the E. coli group (Fig. 2A). These monkeys had 2.079-fold lower (average, 42 ± 24.3 CFU/mL), 31.15-fold lower (average, 11 ± 6.37 CFU/mL), and 70.88-fold lower (average, 3 ± 2.1 CFU/mL) viable bacteria than those in the E. coli control at 30 min, 2 h, and 4 h, respectively, with no viable bacteria detected in the subsequent time points. All of these reductions in bacteremia were found significant (*P *= 0.0001). When we analyzed the bacteremia in rhesus from the Fh15-Fh15-E. coli group, a similar pattern was observed, although there were some differences. In two animals (MA014 and MA035), the bacteremia reached maximal values at 2 h (47 ± 35 CFU/mL), declined quickly at 4 h (4 CFU/mL), and was undetectable thereafter. In the rhesus MA078, the maximal number of viable bacteria was also detected at 2 h (226 CFU/mL), declined to 38 CFU/mL by 4 h, and declined further to 16 CFU/mL and 6 CFU/mL at 6 h and 8 h, respectively. Statistical differences were found between the bacteremia in the Fh15-Fh15-E. coli group compared with that in the E. coli group at 30 min (*P *= 0.0488) and 4 h (*P *= 0.0055). However, no statistical differences were found between the levels of bacteremia in the Fh15-E. coli and Fh15-Fh15-E. coli groups at any of the time points studied. These results demonstrate that the treatment with Fh15 applied 30 min prior to exposure to a lethal live E. coli infusion can efficiently prevent the bacterial replication in blood.

In agreement with the decreasing of bacteremia, the LPS levels in plasma were also notably lower in both experimental groups than those in the E. coli group. In the Fh15-E. coli group, the average concentration of LPS at 30 min was 169.16 ± 29.27 EU/mL, which represents a significant decrease of 1.44-fold of LPS compared with that in the E. coli group (*P* = 0.0067). The concentrations of LPS in this experimental group lowered subsequently, reaching the lowest levels at 8 h (7.3 ± 2.5 EU/mL), which represented a significant diminution (*P* = 0.0042) of 36.60-fold compared with those of the E. coli group (Fig. 2B). In the group Fh15-Fh15-E. coli, the levels of LPS at 30 min were very low but at 2 h had reached a peak (average, 258.33 ± 38.11 EU/mL). Afterward, in two monkeys (MA014 and MA035), the LPS concentrations reduced sequentially until they reached the lowest levels of 29.75 ± 14.65 EU/mL at 8 h, which represented a significant lowering by 8.98-fold compared with that in the E. coli group (*P *= 0.0224). However, in monkey MA078, the LPS concentrations remained consistently high throughout the experiment, with an average concentration of 218.6 ± 17.63 EU/mL. Statistical differences between both experimental groups and the E. coli group were found at 4 h, 6 h, and 8 h (*P < *0.0001). The Fh15-E. coli and Fh15-Fh15-E. coli groups had significantly lower levels of CRP and PCT than the E. coli group (*P *0.0001) (Fig. 2C and D).

### Fh15 suppress the production of proinflammatory cytokines in plasma of septic rhesus macaques.

**FIG 3:**
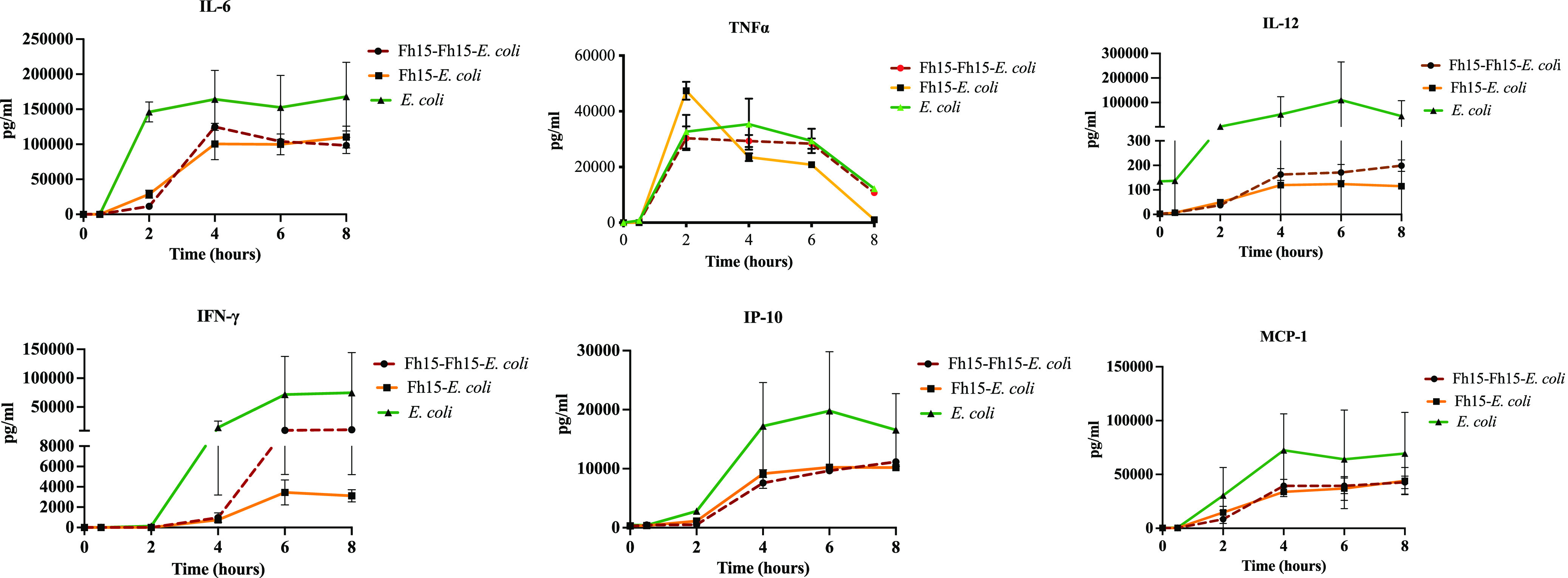
Fh15 tended to decrease the production of proinflammatory cytokines and chemokines in rhesus monkeys undergoing E. coli infection. Plasma samples were collected from rhesus macaques at different time points, as follows: 0 h (baseline), 30 min, 2 h, 4 h, 6 h, and 8 h. E. coli, comprised animals that only received the i.v. infusion with live E. coli (10^10^ CFU/kg body wt.); Fh15-E. coli, comprised animals that received an i.v. infusion containing 12 mg Fh15 followed by the E. coli infection; and Fh15-Fh15-E. coli, comprised animals from the Fh15 group that 3 months later received a second i.v. infusion with 12 mg Fh15 followed by the E. coli infection. Levels of IFN-γ, IL-6, IL-12, TNF-α, MCP-1, and IP10 were measured using Luminex technology. Animals from the Fh15 group do not elicit proinflammatory cytokines (data not shown).

Having demonstrated that the administration of an Fh15 prior to a live bacterial infusion is able to suppress bacteremia and endotoxemia, as well as levels of CRP and PCT induced by E. coli, we proceeded to investigate whether Fh15 could also suppress several proinflammatory cytokine/chemokines that are signatures of inflammation during sepsis. As expected, all animals from the E. coli group developed a strong proinflammatory cytokine storm evidenced by the high levels of IFN-γ, IL-6, TNF-α, IL-12, interferon gamma-induced protein 10 (IP-10), and monocyte chemoattractant protein-1 (MCP-1) throughout the time course. This finding is consistent with the high levels of LPS, CRP, and PCT detected in the plasma of these animals, which are indicative of an ongoing bacterial sepsis. However, all animals from groups Fh15-E. coli and Fh15-Fh15-E. coli had lower concentrations of IL-6, TNF-α, IL-12, and IFN-γ cytokines and lower IP10 and MCP-1 chemokines than monkeys from the E. coli control group at every time point studied (Fig. 3). Unfortunately, due to the large variability between outbred NHPs, levels of cytokines/chemokines among monkeys from a same group were not statistically different. To better appreciate the reduction that produced Fh15 in the levels of these proinflammatory cytokines/chemokines, we determined the fold changes for the decrease in cytokine/chemokine levels by dividing the concentration of each cytokine/chemokine induced by E. coli at every time point by the mean concentration of the cytokine/chemokine induced by the Fh15 treatment, and the result was expressed as a negative value (see Table S3 in the supplemental material). This analysis revealed that animals from the Fh15-E. coli group had 11.28- to 23.97-fold less IFN-γ than the E. coli group at 2 h to 8 h, respectively. The reduction in IFN-γ levels in the Fh15-Fh15-E. coli group ranged from 14.95 to 7.03 at 2 h and 8 h, respectively. IFN-γ is a cytokine classically produced by natural killer (NK) cells and T lymphocytes, which facilitates systemic inflammation during endotoxin-induced shock (34). IL-12, a cytokine naturally produced by dendritic cells (DCs), macrophages, and neutrophil cells in response to antigenic stimulation (35–39), was found to be 20.66- to 386.18-fold reduced in the Fh15-E. coli group and 18.36- to 223.44-fold reduced in the Fh15-Fh15-E. coli group. TNF-α, an inflammatory cytokine produced by macrophages/monocytes during acute inflammation (40), which normally has a peak of secretion about 2 h following the E. coli insult (28) with a further decline later, was also found reduced from 23.34-fold to 11.42-fold in the Fh15-E. coli group and 6.57- to 1.03-fold reduced in the Fh15-Fh15-E. coli group, during the entire time course. IL-6, a cytokine produced by a variety of cells, including macrophages and monocytes during inflammatory process (41), was also found to be 2.33-fold and 1.52-fold reduced at 2 h and 8 h, respectively, in the Fh15-E. coli group as well as 2.31-fold and 1.7-fold reduced in the Fh15-Fh15-E. coli group at 2 h and 8 h, respectively. Similarly, the chemokines IP-10 and MCP-1 were found to be 5.41- to 1.48-fold or 1.76- to 1.63-fold reduced at 2 h and 8 h, respectively, in the Fh15-Fh15-E. coli group compared with the E. coli group (Table S3).

### Effect of bacterial infusion on the innate immune cell population and the counter effect caused by Fh15.

**FIG 4:**
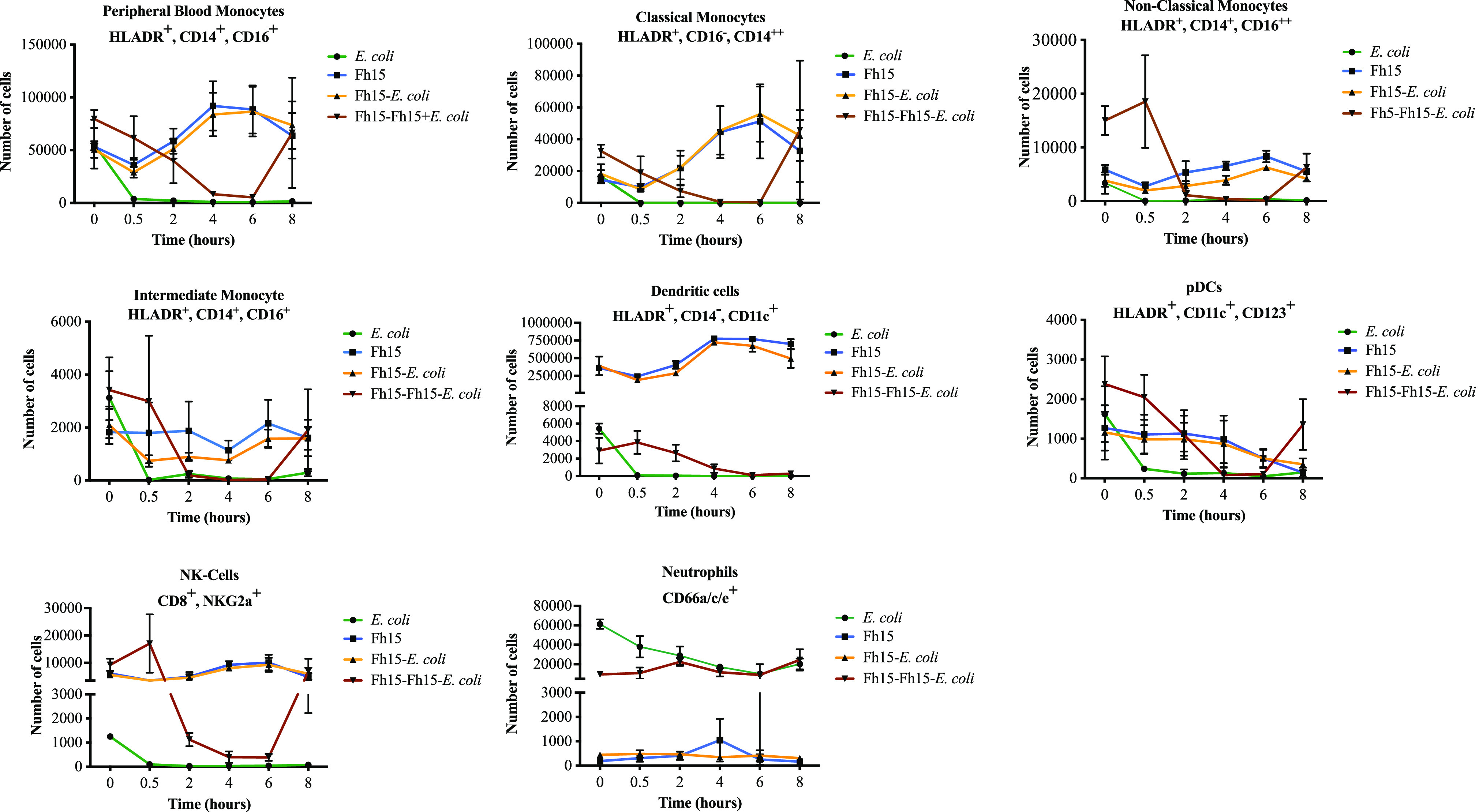
Effect of live E. coli compared with that of Fh15 treatment on the number of innate immune cells in the bloodstream. Graphs represent the number of peripheral blood monocytes, classical monocytes, nonclassical monocytes, intermediate monocytes, dendritic cells, plasmacytoid DCs, natural killer cells, and neutrophils over time in four experimental groups, as follows: E. coli, rhesus macaques (*n* = 3) that received an i.v. isotonic infusion with live E. coli (10^10^ CFU/kg body wt.); Fh15, rhesus macaques (*n* = 3) that received only i.v. infusion with 12 mg Fh15; Fh15-E. coli, rhesus macaques (*n* = 3) that received the Fh15 infusion followed by the E. coli infection; and Fh15-Fh15-E. coli, rhesus macaques (*n* = 3) from the Fh15 that 3 months later received a second infusion with 12 mg Fh15 followed by the E. coli infusion. Whole-blood samples were stained with a cocktail of antibodies for labeling surface markers specific for peripheral monocytes (CD14^+^ and CD16^+^), myeloid and plasmacytoid dendritic cells (CD11c^+^ and CD123^+^), natural killer cells (HLA-DR^+^, CD3^−^, and CD69^+^), and neutrophils (CD66a/c/e^+^). Cells were analyzed by gating using a MacQuant10 instrument. Data were analyzed using FlowJo software. Data represent the average number of cells ± SD of three independent biological samples, with each in duplicate at every time point studied.

When the blood innate immune cell effector populations in the group that received only the E. coli infusion were examined, it was noticed that most of the cell populations had significantly decreased by 30 min following the infusion (*P < *0.0001) and remained at very low levels throughout the full time course of the experiment (Fig. 4). Specifically, peripheral blood monocytes decreased by >14.8-fold compared with the baseline. Classical monocytes, which comprise about 80% to 95% of circulating monocytes and are highly phagocytic (42, 43), had decreased >2,000-fold. Nonclassical monocytes that comprise about the 2% to 11% of circulating monocytes (44) and have proinflammatory behavior, had decreased >100-fold. Intermediate monocytes, which comprise about 2% to 8% of circulating monocytes and have among their functions the production of reactive oxygen species (ROS), antigen presentation, stimulation, and proliferation of T cells and angiogenesis (44), had decreased >130-fold. Dendritic cells (DCs) and plasmacytoid DCs (pDCs), which play pivotal roles in the initiation of innate and adaptive immune response to pathogens (45), decreased >50-fold and > 6-fold, respectively. Natural killer (NK) cells, which make up 5% to 15% of human peripheral blood (46) and play protective roles against both infectious pathogens and cancer (47), decreased >12-fold.

Interestingly, in the group that received only the Fh15 infusion, most of these cell populations increased progressively until reaching maximal levels between 4 to 8 hours. At peak, the amount of the classical monocyte population was 3.5-fold higher than that of the baseline; there were 1.42-fold more nonclassical monocytes and 1.18-fold more intermediate monocytes than those at the baseline. The DC and NK cell populations also were found to be 2.13-fold and 1.64-fold more abundant than those in at baseline, respectively. Although the number of cells decreased slightly after peak, all cell populations remained at high levels until the end of the study. The pDCs and neutrophils remained at levels relatively like those at the baseline, although the trend of pDCs was to decline sequentially. Importantly, a dynamic almost identical to that described for Fh15 was observed within animals from the Fh15-E. coli group. The Fh15-Fh15-E. coli group had a different dynamic of innate immune cells in which the amount of all monocyte populations, DCs, and NK cells declined sequentially until they reached the lowest levels between 4 to 6 hours and then increased notably at the end of the procedure (Fig. 4; see Table S4 in the supplemental material).

## DISCUSSION

Our previous *in vitro* studies using THP1-Blue CD14 cells and mouse bone marrow-derived macrophages (BMDMs) demonstrated that Fh15 can suppress the NF-κB activation induced by different Gram-negative and Gram-positive bacterial extracts and suppress a number of the LPS-induced proinflammatory cytokines, respectively (31). Moreover, *in vivo* studies using a toxemia mouse model of sepsis demonstrated that Fh15 could be useful as a prophylactic and therapeutic drug to prevent the dissemination of the cytokine storm in the animals that received Fh15 (25, 48). Based on these antecedents and as a proof of concept, the current study aimed to ascertain whether Fh15 could be useful for limiting the lethal inflammatory responses and bacterial dissemination caused by an i.v. infusion with live E. coli in rhesus macaques.

Fh15 is an immunogenic molecule capable of inducing a strong antibody response when it is injected subcutaneously (s.c.) in rabbits or mice emulsified in adjuvant (23, 49). Hence, rabbits and mice infected with *F. hepatica* also develop antibodies against Fh15 (23, 49). Therefore, it was not surprising that all rhesus macaques that received the Fh15 infusion in the present study have detectable antibody against Fh15 3 months later. However, what was surprising was the observation that these same animals had not only antibodies but also higher levels of antigenemia. Although in the Fh15 group the main contributor to the antigenemia was MA035, in the Fh15-Fh15-E. coli group, the three monkeys (MA078, MA014, and MA035) contributed similarly to the antigenemia. To rule out any technical mistake, these determinations were repeated three times in different days with similar results. Therefore, although at present we have no explanation for the higher antigenemia in the Fh15-Fh15-E. coli compared with that in other groups, it is possible to think that the pharmacokinetics of Fh15 could be affected by the presence of antibodies, undetected assay interferences, or other mechanisms.

The detection of antibodies against Fh15, concurrently with circulating Fh15, suggests that the antibodies elicited against Fh15 had low affinity and did not attach strongly enough to the antigen; otherwise, the sandwich ELISA would not have detected circulating Fh15. The production of antibodies of low titer and low affinity often occurs when the exposure to the antigen has been discontinued and fail the selection of clones that produce an antibody of high affinity (50). The rationale to include in the present study an experimental group that has antibodies specific against Fh15 was precisely to determine whether these anti-Fh15 antibodies may block the ability of Fh15 to exert its anti-inflammatory function. In this regard, it was interesting to observe that animals with similar levels of anti-Fh15 antibodies (Fh15-Fh15-E. coli group) had a similar ability to those that were naive to Fh15 (Fh15-E. coli) to prevent bacterial replication, reduce the concentration of plasma LPS, and suppress the production of CRP and PCT (Fig. 1), suggesting that at least these functions were not impaired by the presence of antibodies. In humans undergoing sepsis or septic shock, CRP is known to activate the complement system and increase antigen presentation. Also, it has been correlated with an increased organ dysfunction, longer hospital intensive care unit (ICU) stay, and mortality (51). PCT in the ICU is used as a biomarker for survival, with lower levels resulting in lower sequential organ failure assessment (SOFA) scores and better prognosis in septic patients (52).

The observation that two monkeys (6R1 and 0R5) that received only the E. coli infusion had bacteremia by only 4 hours is an expected event. The rapid decline of bacteremia in these monkeys indicates that bacteria were unable to colonize and replicate inside these animals and were lysed by the complement system, as it has been reported under experimental conditions where high bacteria inoculum are used to induce sepsis (53). Since all animals received the same bacterial dose adjusted to the body weight (wt.), the persistent detection of bacteremia in one monkey (CB22) from the E. coli group suggests failure in the efficiency of the complement system to lyse bacteria. An important observation of the present study was the capacity of Fh15 to suppress bacteremia. The mechanism that uses Fh15 to suppress the bacteremia remains unknown. However, we have speculated that Fh15 could be capable of inducing immune cells to release extracellular DNA traps (ETs) to trap bacteria. This process termed etosis is currently under study in our laboratory. It is a distinct process of cell death and has been reported to occur in several immune cells, including neutrophils (54), eosinophils (55), mast cells (56), and monocytes/macrophages (57) from humans or mice.

Another interesting observation of the present study was the abrupt and sustained decrease in the blood innate immune cells during the 8 hours following the E. coli infusion (Fig. 3). Blood monocytes (including classical, nonclassical and intermediate monocytes), NK cells, and DCs are key during the early phase of endotoxemia because they are not only responsible for maintaining vascular homeostasis but also highly responsible for patrolling the bloodstream to recognize and phagocytize invading pathogens, resulting in the secretion of proinflammatory cytokines, such as IL-1β, TNF-α, IL-6, and IL-8 (58–60). The “disappearance” of immune cells from the bloodstream in rhesus macaques from the E. coli group could be a logical consequence after an inflammatory insult since immune cells migrate to secondary lymphoid organs to maturate, differentiate, and present the antigen to the naive T cells (61). This observation is consistent with a similar phenomenon fully documented that occurs in mice exposed to lethal intraperitoneal doses of LPS in which large peritoneal macrophages (LPMs), the macrophage population more abundant at steady state, “disappear” from the peritoneal cavity at the beginning of the LPS insult (25, 62). In that regard, we have demonstrated that Fh15 shifts the LPM dynamics by not only preventing the disappearance of LPMs but also augmenting this cell population in the peritoneal cavity (25). The observation that Fh15 alone or in the presence of E. coli can promote the persistence of innate immune cells in the bloodstream during the early phase of the endotoxemia leads us to suggest that a primary modulatory mechanism of Fh15 would be based on generating an immunological environment compatible with the homeostasis or a prolonged steady state even in the presence of inflammatory stimuli. However, based on the results shown in Fig. 4, the ability for Fh15 to increase the innate immune cell population seems to be partially abrogated in the rhesus macaques from group Fh15-Fh15-E. coli. The decline in the number of cells accounted for not only the monkey that had higher endotoxemia and bacteremia (MA078) but also the other two monkeys. However, it should be noticed that the reduction of cell populations in this experimental group was sequential and not abrupt, which occurred in animals that received only E. coli, and at the end of the experimental window, all cell populations had again increased. Since all monkeys in the Fh15-Fh15-E. coli group had in common the presence of anti-Fh15 antibodies, we speculate that the antibodies could be responsible for modifying the cell dynamics. However, considering that in these same monkeys the presence of antibodies does not seem to affect the decrease of bacteremia, endotoxemia, CRP, PCT, or the ability for Fh15 to reduce the proinflammatory cytokine/chemokines (Fig. 2), it should not be ruled out that other immunologic factors, unknown so far, could have influenced this result.

The observation that Fh15 alone does not induce IL-12, IL-6, IFN-γ, TNF-α, IP-10, or MCP-1 is consistent with our previous studies in which native or recombinant *F. hepatica* FABPs are unable to elicit inflammatory responses (25, 31, 48). Importantly, despite the large variability among the cytokine/chemokine concentrations for each monkey in the experimental groups, it was possible to observe a notable lowering in the concentrations of IL-12, IL-6, IFN-γ, TNF-α, IP-10, or MCP-1 in the plasma of animals that received the Fh15 infusion compared with those that received only the E. coli infusion. The lack of statistical differences in these determinations could be due to several factors acting simultaneously. Some possible explanations could be the following: the immunological variability typical in outbreeds subjects, the low number of animals per group, and the lack of an appropriate adjustment in the amount of Fh15 administered to monkeys according to their body weight made it difficult to determine the real behavior of data and identify outlier subjects. It should be highlighted that the low dose of Fh15 administered to monkeys was based on the amount of purified Fh15 availability rather than a rigorous dose-dependent experiment to select the best Fh15 concentration that maximizes the results. If we consider that the groups of monkeys in our experiment were weighing 6.4 to 9.66 kg and all received the same Fh15 dose (12 mg), it meant that some animals received 1.87 mg/kg of body weight of Fh15, whereas others received a dose equivalent to 1.24 mg/kg of body weight. Overall, the amount of Fh15 administered to monkeys in the present study was lower than that applied intraperitoneally to BALB/c mice (50 μg per mouse) (25), which assuming that all mice weight ∼20 g, would be equivalent to 2,500 μg/kg of body weight. Moreover, as mentioned above, the observation that the group that had anti-Fh15 antibodies (Fh15-Fh15-E. coli) reduced the concentrations of plasma IL-12, IL-6, IFN-γ, TNF-α, IP-10, or MCP-1 in a similar magnitude to those observed in the Fh15-E. coli that does not have anti-Fh15 antibodies indicates that the presence of anti-Fh15 antibodies does not impair the capacity of Fh15 to suppress the cytokine storm. This conclusion is consistent with results of a previous experiment performed with THP1-Blue CD14 cells in which it was demonstrated that the presence of anti-Fh12 antibodies does not abrogate the capacity of Fh15 to suppress the LPS-induced TLR4 stimulation (31). The lowering of proinflammatory cytokines induced by Fh15 is also in agreement with other reports in which *F. hepatica* infection has been shown to suppress Th1 responses in concurrent bacterial infections (14, 63). These findings prelude a promising therapeutic outcome in a rhesus preclinical sepsis model and support the therapeutic potential of Fh15 as an anti-inflammatory agent.

Three novel issues stand out from the results reported in this communication. We found that a small recombinant molecule belonging to the *F. hepatica* fatty acid binding protein (Fh15) administered intravenously in rhesus macaques prior to an infusion with lethal amounts of live E. coli (i) significantly reduces bacteremia and subsequently suppresses the levels of LPS in plasma and reduces the production of CRP and PCT, which are key signatures of inflammation and bacterial infection, respectively; (ii) notably reduced the production of proinflammatory cytokines and chemokines; and (iii) prevented the immune cell disappearance from the bloodstream. Instead, Fh15 promoted the increase of innate immune cell populations in blood, a phenomenon that seems to be like those observed for LPMs in the peritoneum of septic mice. This result could suggest a role for Fh15 in promoting a prolonged steady state or homeostasis in rhesus macaques even in the presence of inflammatory stimuli.

The small number of samples and the lack of an adequate adjustment in the Fh15 dose based on body weight, which could have contributed to the variability in some results, limit the present study. However, despite these limitations, the results of this communication showing that a single prophylactic dose of Fh15 can suppress the bacteremia, the levels of endotoxemia, the levels of acute-phase proteins, and a large panel of proinflammatory cytokines/chemokines during the early phase of sepsis are highly promising and could be translated to a better prognosis in this rhesus macaque preclinical sepsis model. More studies are in progress to determine the mechanism by which Fh15 promotes bacterial killing and clearance and its ability to decrease inflammatory markers. Importantly, more preclinical studies are underway to define the optimal Fh15 dose for therapeutic efficacy as well as determine its therapeutic potential after a live E. coli infusion or after ongoing sepsis.

## MATERIALS AND METHODS

### Ethics statement.

This study was performed in accordance with the Guide for the Care and Use of Laboratory Animals (National Research Council [US], 2011) and was approved by the Institutional Animal Care and Use Committee of the University of Puerto Rico‐Medical Sciences Campus (protocol number 7870116).

### Animals.

Nine healthy adult male (6 to 7 years old) rhesus macaques (Macaca mulatta), weighing 6.4 to 9.66 kg (8.1 ± 1.08), were kindly donated by the Caribbean Primate Research Center at the University of Puerto Rico-Medical Sciences Campus. Prior to inclusion in the experiment, the monkeys received a physical examination and were tested for hematological, serological, and microbiological abnormalities. Only the animals confirmed healthy by a veterinarian were included into the experiment.

### High-throughput protein expression and purification of Fh15.

cDNA encoding a full-length fatty acid binding protein from *F*. *hepatica* (Fh15) (GenBank accession number M95291.1) (64) was synthesized and cloned into the pT7M vector and expressed as a fusion protein with a His tag (6×His) at the amino terminal in a novel bacterial expression system, using Bacillus subtilis (Genscript USA). This model offers more advantages than our previously optimized Fh15 expression system in E. coli (31). B. subtilis is a nonpathogenic Gram-positive bacterium that does not produce LPS. Furthermore, it is not codon biased, grows faster, and has a higher secretory capacity than the customary bacterial expression in E. coli. *Bacillus* strain 7024E was transformed with a recombinant plasmid. A single colony was inoculated into terrific broth (TB) medium; the culture was incubated at 37°C; and when the optical density at 600 nm (OD_600_) reached about 1.2, the protein overexpression was induced with isopropyl-β-d-thiogalactopyranoside (IPTG) at 37°C for 4 h. Cells were harvested by centrifugation, and cell pellets were resuspended with lysis buffer followed by sonication. After centrifugation, the precipitate was dissolved using a denaturing agent. Fh15 was purified from inclusion bodies by one-step purification using an Ni column. Fh15 was stabilized in 1× phosphate-buffered saline (PBS; pH 7.4) containing 10% glycerol and 0.5 M NaCl and sterilized via a 0.22-μm filter. A Western blot with a mouse anti-histidine tag monoclonal antibody (Genscript; catalog [cat.] number A00186) was used to confirm the purity of the purified protein (see Fig. S1 in the supplemental material). Purified Fh15 had endotoxin levels lower than 0.2 EU/mg measured by a limulus amebocyte lysate (LAL) endotoxin assay kit (Bioendo; cat. number KC64T). The protein concentration of Fh15 (3.86 mg/mL) was determined by the Bradford method with bovine serum albumin (BSA) as a standard (ThermoFisher; cat. number 23236).

### Anti-Fh15 polyclonal antibody and conjugation.

A polyclonal antibody against Fh15 was produced in New Zealand White female rabbits by subcutaneous (s.c.) injections of 200 μg of protein mixed with an equal volume of complete Freund’s adjuvant in the first injection and incomplete Freund’s adjuvant in the boost injections as described previously (48). The antiserum had antibody titers of ∼1:100,000 when it was titrated by indirect ELISA against the Fh15. Anti-Fh15 polyclonal IgG was purified by affinity chromatography using 5/5/HiTrap Protein-A HP (GE Healthcare, Piscataway, NJ). The resulting IgG was conjugated with horseradish peroxidase (HRP; Abcam, UK; ab102890) and used to develop a sandwich ELISA to detect the presence of circulating Fh15 in the blood of rhesus macaques used in this study.

### Escherichia coli culture.

The Escherichia coli 086a: K61 serotype used to induce sepsis was purchased from American Type Culture Collection (ATCC 33985). Two days prior to each experiment, a fresh glycerol stock was plated on Luria Broth agar and cultured for 20 hours at 37°C. The next day, a single colony was cultured in 300 mL of Luria Broth for 16 to 18 hours at 37°C, until it reached a concentration of 10^10^ CFU/mL. Afterward, to remove free LPS, the culture was harvested and washed twice with endotoxin-free PBS (0.01 M phosphate-buffered saline pH 7.4). The pellet was resuspended in 50 mL of endotoxin-free PBS and administered to animals.

### Animal preparation and intravenous infusion administration.

Monkeys were fasted overnight prior to the experiment and sedated the next day with ketamine-hydrochloride (100 mg/mL) intramuscularly (i.m.) (10 mg/kg) (Akorn-Lake Forrest, IL). Afterward, they were transported to the surgical suite and anesthetized with isoflurane gas (Akorn Inc, Lake Forest, IL) via a facemask. Afterward, monkeys were intubated with 3.0- to 3.5-mm endotracheal tubes for anesthesia maintenance and were kept intubated for the entire 8-hour experimental period allowing them to breathe on their own. A 20-gauge intravenous catheter (Nipro-Osaka, Japan) was inserted in the lateral saphenous to inject the infusions prepared in isotonic saline containing 2.5% dextrose at a rate of 3.3 mL/kg/h to compensate for fluid loss. To avoid causing discomfort, we allowed the animals to remain under anesthesia during the entire experimental window of 8 hours. Monkeys were connected to a Bionet monitor that measured physiological parameters, such as BT, HR, RR, and MAP, every 10 minutes. The monkeys remained under constant monitoring by veterinary staff during the complete time course of the experiment.

The bacterial infusion consisted of a 50-mL isotonic saline solution containing 10^10^ CFU/kg body weight (wt.) of live E. coli, which was applied at a constant rate infusion (CRI) of 0.42 mL/min over 2 h. This dose was determined to be a lethal dose for these subjects as reported elsewhere (65). There are no previous studies with rhesus macaques that have been injected i.v. with proteins. Thus, a dose of 12 mg/monkey was used based on the amount of Fh15 available for the study. The 12-mg Fh15 dose was dissolved in a 5-mL isotonic saline and applied at a constant rate infusion of 0.25 mL/min over 20 minutes. The Fh15 dose was equivalent to 1.24 to 1.87 mg/kg of body weight (average, 1.48 mg/kg body wt.).

### Experimental groups.

Rhesus macaques were allotted randomly into four experimental groups designated E. coli, Fh15, Fh15-E. coli, and Fh15-Fh15-E. coli. The group designated E. coli was a positive-control group for sepsis that received only the bacterial isotonic infusion. Following E. coli infusion, animals were monitored for 8 h and then euthanized. The group designated Fh15 received only an isotonic infusion of 5 mL containing 12 mg Fh15 for 20 min and was monitored for 8 h; they were allowed to recover from anesthesia and returned to their original cages for 3 months. The group named Fh15-E. coli received the isotonic infusion of Fh15 for 20 min followed immediately by an E. coli infusion and were euthanized at 8 h of experimentation. The group named Fh15-Fh15-E. coli was comprised of monkeys in the Fh15 group that 3 months later received a second infusion of Fh15 and were infected with E. coli. After being infected with E. coli, monkeys were euthanized at 8 h as described above (see Fig. S2 in the supplemental material). Due to limitations of space and resources for monitoring more than 1 monkey at a time, no animals of the experimental group were associated temporally. Every single monkey was dosed separately under identical experimental conditions. Monkeys from the E. coli group were dosed first (one monkey per day) followed by monkeys that received Fh15 and Fh15-E. coli. The monkeys from Fh15-Fh15-E. coli group were dosed last (3 months after the first Fh15 treatment).

Blood samples of 5 mL were taken from the femoral vein of all animals using a 20-gauge needle and were collected into heparinized tubes (BD Vacutainer plastic blood collection tubes; Fisher Scientific) at 0 min, 30 min, 2 h, 4 h, 6 h, and 8 h of experimentation. Blood samples were centrifuged at 10,000 rpm for 10 min, and the plasma was collected and stored at −20°C in aliquots of 500 μL until further use. Prior to centrifugation, aliquots of 500 μL and 150 μL from each blood sample were allotted to determine bacteremia levels and quantify immune cell populations by flow cytometry, respectively. After the completion of the experimental window, monkeys were euthanized with a pentobarbitol solution of 390 mg/mL (Med-Pharmex, Pomona, CA) according the AVMA Guidelines for the Euthanasia of Animals (66). A postmortem examination of all monkeys was conducted immediately after they were euthanized. Gross necropsy was performed and animals were examined for any abnormalities.

### Bacteremia and endotoxin level assessment.

To assess the number of viable bacteria, 500 μL of whole blood was diluted 1:1 with sterile 1× PBS, spread onto a Luria Broth (LB) agar plate, and cultured for 20 h at 37°C. Colonies were counted and adjusted by the dilution factor. The presence of circulating levels of LPS was determined using the Pierce endotoxin quant kit following the manufacturer’s instructions (Thermo Research Scientific, USA; A39553).

### C-reactive protein (CRP) and procalcitonin (PCT) concentration determination.

Plasma samples from each time point were tested for the quantification of CRP and PCT, which are characteristic inflammatory biomarkers and a hallmark of bacterial infection, respectively. Quantification of CRP levels was performed at a private clinical laboratory (Martin Inc., Bayamón, PR) using an Architect c8000 clinical chemical analyzer (Abbott, Illinois, USA). The PCT assay was performed using a Human Procalcitonin ELISA kit following manufacturer’s instructions (Abcam, UK; ab100630).

### Flow cytometry.

Flow cytometry analyses were done to determine the effect of Fh15 on innate immune effector cells. A total of 150 μL of anticoagulated peripheral blood was stained with a surface antibody cocktail for 30 minutes at 4°C in the dark. Two antibody cocktails were used for the analysis, which are listed in detail in the Table S1 in the supplemental material. After cells were stained, lysis buffer (BD Biosciences, USA) was used to lyse the red blood cells and fix the samples for 10 minutes in the dark. Cells were washed with fluorescence-activated cell sorter (FACS) buffer three times. Samples were stored at 4°C in the dark until the next morning for data acquisition using a MacQuant10 instrument (Miltenyi Biotec, USA). Data were analyzed using FlowJo 10 (BD Biosciences). Our gating strategy for monocytes based on their expression of CD14 and CD16 as described elsewhere (44) included the following: classical monocytes (CD14^++^ and CD16^−^), nonclassical monocytes (CD14^+^ and CD16^++^), and intermediate monocytes (CD14^+^ and CD16^+^). Neutrophils were gated based on their expression of HLA-DR^+^, CD3^−^, and CD66 a/c/e^+^; dendritic cells (DCs) and plasmacytoid DCs based on their expression of HLA-DR^+^, CD11c^+^, CD3^−^, and CD123^+^; and NK cells based on their expression of CD3^−^, HLA-DR, CD8^+^, and NKG2a^+^.

### Sandwich ELISA for detecting circulating Fh15 in the plasma.

A sandwich ELISA was optimized to detect circulating Fh15 in plasma from animals that received an i.v. isotonic infusion containing Fh15. The protocol followed was similar to those developed previously to detect circulating antigens in human samples (67), but we used the purified rabbit anti-Fh15 IgG and the anti-Fh15 IgG-HRP conjugate described above at optimal concentrations that were determined by checkerboard titration. The anti-Fh15 IgG antibody was used as a capturing antibody. It was assayed in duplicates using dilutions ranging among 0.25 to 20 μg/mL in coating buffer (0.05 M carbonate-bicarbonate [pH 9.6]). Disposable polystyrene 96-well plates (Costar, Corning, NY) were coated with 100 μL/well of each capturing antibody dilution. After an overnight incubation at 4°C in a humid chamber, the plate was washed three times with phosphate-buffered saline with Tween 20 (PBST) and blocked with 5% skimmed milk-PBST (300 μL/well) for 1 hour at 37°C in a humid chamber. After the blocking solution was removed, undiluted plasma samples (100 μL/well) were added to the plate and incubated for 1 h at 37°C. Afterward, the plates were washed three times with PBST. Anti-Fh15 IgG-HRP used as a detecting antibody was prepared at different dilutions ranging among 1:1,000 and 1:3,000 in PBST and added to the plates (100 μL/well), and the incubation was prolonged for 30 minutes at 37°C in a humid chamber. After another washing step, the substrate solution (50 mL 0.05 M citrate-phosphate [pH 5.0] + 10 mg *o*-phenylenediamine + 10 μL H_2_O_2_) was added to each well (100 μL/well) and the plate was incubated at room temperature for 15 minutes in the dark. The reaction was stopped by adding 50 μL/well of 10% HCl, and the optical density (OD) was measured at 490 nm using a SpectraMax M3 instrument (Molecular Devices, LLC, USA). A standard curve was generated by adding Fh15 to a negative baseline plasma sample with different concentrations of Fh15 ranging from 1 μg/mL to 61.03 pg/mL. The OD at 490 nm of the spiked negative plasma sample was plotted against its known antigen protein concentration. The standard curve had an r^2^ of 0.99 using a 4 Parameter Logistic (PL) analysis. By use of the standard curve, the ODs of plasma from animals used in the sepsis model were transformed into antigen concentrations (μg/mL).

### Plasma cytokine and chemokine level determination.

Levels of plasma proinflammatory cytokines IL-6, IL-12p70, TNF-α, and IFN-γ and chemokines MCP-1 and IP-10 were determined using an NHP customized multiplex system (R&D Systems, Minneapolis, USA; catalog number FCSTM21) following the manufacturer’s instructions. Briefly, the assay uses magnetic microparticles precoated with analyte-specific antibodies, which are embedded with fluorophores at set ratios for each unique microparticle region. Microparticles, standards, and plasma samples are pipetted into wells and the immobilized antibodies bind the analytes of interest. Plasma samples require a 2-fold dilution with a calibrator diluent provided in the kit. After any unbound substances are washed away, a biotinylated antibody cocktail specific to the analytes of interest is added to each well. Following a wash to remove any unbound biotinylated antibody, a streptavidin-phycoerythrin conjugate (streptavidin-PE), which binds to the biotinylated antibody, is added to each well. After a final washing step to remove unbound streptavidin-PE, the microparticles are resuspended in buffer and read using a Magpix instrument (Luminex, USA) and analyzed with the Bio-Plex Data Pro software (Bio-Rad, Hercules, CA).

### Statistical analysis.

Data were analyzed by using two-way ANOVA and unpaired Student’s *t* test using GraphPad Prism software (version 8). Data differences were considered significant at a *P* value of <0.05.
