# Supplementary material for: Recombinant Fasciola hepatica Fatty Acid Binding Protein as a Novel Anti-Inflammatory Biotherapeutic Drug in an Acute Gram-Negative Nonhuman Primate Sepsis Model
Source: Microbiol Spectr. 2021 Dec 22;9(3):e01910-21. doi: 10.1128/Spectrum.01910-21 (PMC8694124; doi:10.1128/Spectrum.01910-21)
Supplement: SUPPLEMENTAL FILE 1 — Supplemental material. Download SPECTRUM01910-21_Supp_1_seq8.pdf, PDF file, 1.0 MB [file spectrum01910-21_supp_1_seq8.pdf]

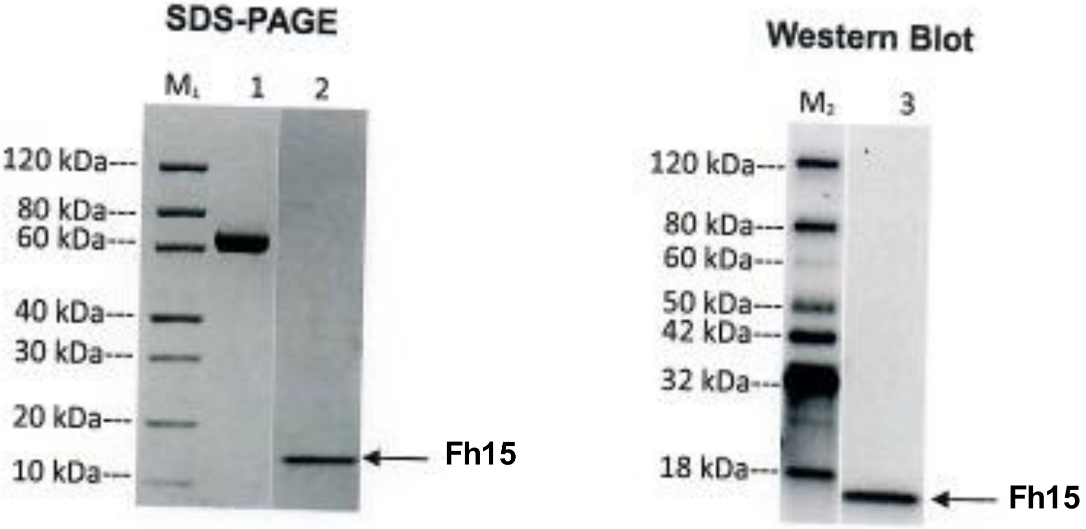

Figure-1S. Purity of Fh15. Recombinant Fh15 was successfully expressed within *Bacillus subtillis* as fusion protein with 6His-Tag at amino terminus. SDS-PAGE and Western Blot were used to assess the purity of the protein. Lane-1 represents a BSA (2µg) as protein control, lane-2 represents Fh15 (2µg) under reducing conditions and lane-3 represents Fh15 (2µg) under reduced conditions incubated with a mouse antibody against the histidine tag.

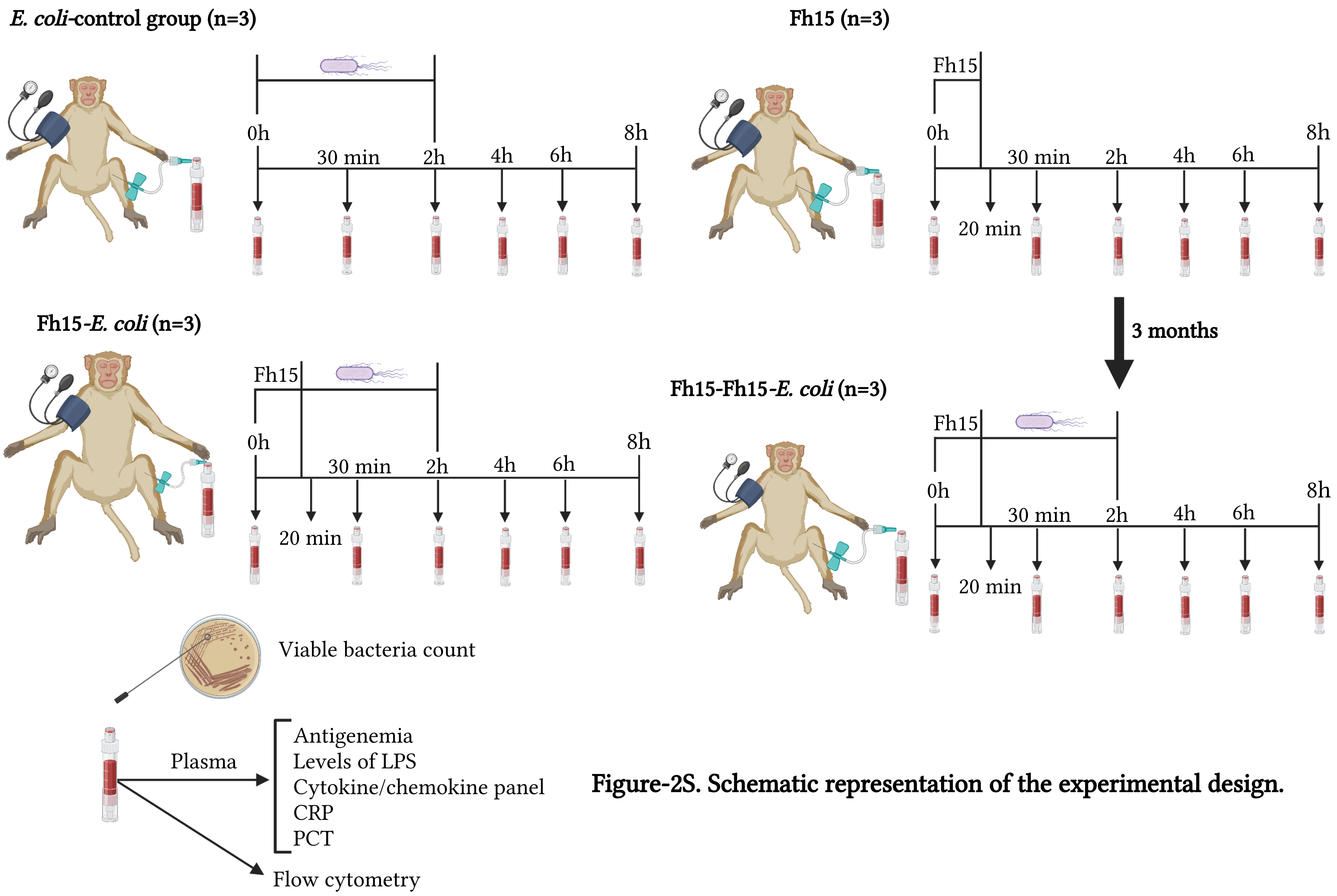

Figure-2S. Schematic representation of the experimental design.

Figure 1S. Purity of Fh15. Recombinant Fh15 was successfully expressed within *Bacillus subtillis* as fusion protein with 6His-Tag at the amino terminal. SDS-PAGE and Western Blot were used to assess the purity of the protein. Lane-1 represents a BSA (2µg) as protein control, lane-2 represents Fh15 (2µg) under reducing conditions and lane-3 represents Fh15 (2µg) under reduced conditions incubated with a mouse antibody against the histidine tag.

Figure 2S. Schematic representation of the experimental design. Diagram represents the experimental design performed with rhesus macaques. Experimental groups (n=3) comprised naïve male rhesus average weighting ~ 8.46kg. Animals were allotted into four groups. The control group termed *E. coli*, only received an i.v. isotonic infusion (50-ml) containing a lethal dose of live *E. coli* (10^{10} CFU/kg body wt.). This infusion was administered by 2h at a flow rate ~0.416ml/min. Another group termed Fh15 only received the isotonic infusion (5-ml) containing 12mg Fh15. This infusion was administered during 20 min at a flow rate ~0.25ml/min. The group termed Fh15-*E. coli* received first the Fh15-infusion followed by the *E. coli*-infusion. The group termed Fh15-Fh15-*E. coli* was comprised by the same animals from Fh15 group, which 3 months after receiving the first Fh15 infusion were returned to the experiment to receive a second Fh15-infusion + *E. coli* infusion. Blood samples were collected at baseline (0 min), 30 min, 2h, 4h, 6h and 8h. All animals that received the *E. coli* infusion were euthanized at 8h. Blood samples collected were used for determining levels of bacteremia and then staining with two antibody cocktails specific for cell markers of innate immune cell populations. These samples were analyzed by flow cytometry. Plasma recovered from the blood samples were used for measuring levels of LPS, antigenemia, CRP, PCT and cytokines/chemokines. Figure was created in BioRender.com

Table-1S. Antibodies cocktail used in the flow cytometry analysis				
**Cocktail-1 (NK-cells)**				
**Cell Marker**	**Labeling**	**Source**	**Catalog Number**	**References**
**CD3**	PerCP-CY5.5	BD	552851	Carter, D.L. et al. 1999 (Cytometry, 37 (1): 41-50)
**NKG2a**	FITC	Miltenyi	130-113-565	Rueda, C. et al. 2016 (J. Immunol. 196 (9): 3706-3715)
**HLA-DR**	VIOGREEN	Miltenyi	130-111-795	Edwards, J.A. et al 1986 (J. Immunol. 137 (2): 490-497
**CD8**	PE	Biolegend	344706	Wakeley, M.E. et al. 2020 (J. Surg. Res. 245:610
**CD69**	APC/Cy7	Biolegend	310914	Ahmed R. et al. 2019 (Cell, 177 (6): 1583-1599
**CD20**	PacBlue	Biolegend	302328	Matzen S.M.H. et al 2018 (Health Sci. Rep. 1: e90)
**Cocktail-2 (MO and DCs)**				
**CD123**	APC	Biolegend	306012	Halim, T.Y.F. et al. 2018 (Immunity, 48 (6):1195-1207)
**CD3**	PerCP-CY5.5	BD	552851	Carter, D.L. et al. 1999 (Cytometry, 37 (1): 41-50)
**HLA-DR**	VIOGREEN	Miltenyi	130-111-795	Edwards, J.A. et al 1986 (J. Immunol. 137 (2): 490-497
**CD11c**	PE/Cy7	Biolegend	337216	Fournier, N. et al. 2018 (MAbs 10: 651-663)
**CD14**	FITC	Biolegend	325604	Walk, J. et al 2019 (Nat Commun. 10:874)
**CD16**	PacBlue	Biolegend	980106	Stroncek, D.F. et al. 1991 (Blood 77:1572-1580)
**CD66a/c/e**	PE	Biolegend	342304	Magri G. et al. 2017 (Immunity, 47: 680)

**TABLE-2S. Main vital signs monitored in *Rhesus macaques* during the entire 8 hours experimental course**				
**Experimental Group**	**ID**	**Physiological Parameter**	**Baseline Value**	**Value at 8h of *E. coli* infusion**
		Body Temperature (˚C)	35.9	38.6
E. coli	6R1	Heart Rate (bpm)	108	192
		Mean Arterial Pressure	58	25
		Respiratory Rate (rpm)	22	29
		Body Temperature (˚C)	37.2	36.1
E. coli	0R5	Heart Rate (bpm)	160	192
		Mean Arterial Pressure	51	49
		Respiratory Rate (rpm)	21	15
		Body Temperature (˚C)	36.3	37
E. coli	CB22	Heart Rate (bpm)	124	172
		Mean Arterial Pressure	31	71
		Respiratory Rate (rpm)	16	24
		Body Temperature (˚C)	35.7	36.4
Fh15	MA078	Heart Rate (bpm)	111	130
		Mean Arterial Pressure	50	55
		Respiratory Rate (rpm)	21	21
		Body Temperature (˚C)	36.8	35.9
Fh15	MA014	Heart Rate (bpm)	148	130
		Mean Arterial Pressure	78	68
		Respiratory Rate (rpm)	14	24
		Body Temperature (˚C)	37.2	37.2
Fh15	MA035	Heart Rate (bpm)	140	142
		Mean Arterial Pressure	53	70
		Respiratory Rate (rpm)	25	30
		Body Temperature (˚C)	35.4	36.3
Fh15-Fh15-*E. coli*	MA078	Heart Rate (bpm)	112	128
		Mean Arterial Pressure	51	49
		Respiratory Rate (rpm)	13	18
		Body Temperature (˚C)	33.4	34
Fh15-Fh15-*E. coli*	MA014	Heart Rate (bpm)	120	164
		Mean Arterial Pressure	33	20
		Respiratory Rate (rpm)	23	20
		Body Temperature (˚C)	35.6	34.6
Fh15-Fh15-*E. coli*	MA035	Heart Rate (bpm)	123	108
		Mean Arterial Pressure	41	21
		Respiratory Rate (rpm)	20	5
		Body Temperature (˚C)	35.9	37.2
Fh15-*E. coli*	8Y4	Heart Rate (bpm)	102	160
		Mean Arterial Pressure	44	23
		Respiratory Rate (rpm)	22	14
		Body Temperature (˚C)	35.6	35.6
Fh15-*E. coli*	0Y2	Heart Rate (bpm)	85	178
		Mean Arterial Pressure	55	10
		Respiratory Rate (rpm)	22	22
		Body Temperature (˚C)	36.1	34.8
Fh15-*E. coli*	IZ8	Heart Rate (bpm)	114	156
		Mean Arterial Pressure	36	24
		Respiratory Rate (rpm)	21	21

**Table-3S.** Fold changes in cytokine/chemokine levels as treatment-related decreases from untreated controls								
**Time (hours)**	***E. coli***		**Fh15-*E. coli***			**Fh15-Fh15-*E. coli***		
	**Mean**	**± SD**	**Mean**	**± SD**	**Fold change***	**Mean**	**± SD**	**Fold change***
**IFNγ**								
0.5h	0	0	0	0	0	0	0	0
2h	137.57	117.7	12.19	4.73	-11.28	9.2	0.001	-14.95
4h	14454.63	11243.36	745.1	35.09	-19.39	974.41	471.23	-30.67
6h	71537.54	66320.46	3451.18	1223.09	-20.72	9587.83	3301.98	-7.46
8h	74824.58	69320.46	3121.45	601.49	-23.97	10643.16	3421.05	-7.03
**IL-6**								
0.5	170.58	68.58	73.15	32.77	-2.33	73.78	28.53	-2.31
2h	146204.65	14128.35	28876.59	5721.59	-5.06	11597.51	4148.98	-12.6
4h	164291.65	41362.35	100554.64	22592.85	-1.63	124881.35	5002.85	-0.11
6h	152573.7	45750.3	99850.77	14896.33	-0.153	104109.4	23.0	-1.46
8h	168073.7	49101.3	110231.42	15726.28	-1.52	98478.78	11487.92	-1.7
**IL-12**								
0.5h	137.39	0.001	6.65	0.009	-20.66	7.48	5.82	-18.36
2h	1717.03	1637.96	49.74	8.39	-34.52	37.63	6.16	-45.62
4h	51350.63	5123.37	119.43	8.81	-429.9	162.76	17.11	-315.49
6h	109921.18	10980.82	123.86	0.12	-887.4	171.22	23.41	-641.98
8h	44414.81	4428.19	115.01	8.84	-386.18	198.77	16.78	-223.44
**TNFα**								
0.5h	764.11	125.11	32.73	8.31	-23.34	116.36	111.46	-6.57
2h	32698.51	6066.49	47363.74	3232.83	-0.69	25336.86	714.95	-1.29
4h	35388.3	9174.7	23519.15	1420.85	-1.5	29323.96	2169.04	-16.31
6h	29361.02	4367.02	20829.645	480.67	-1.40	28375.92	1847.37	-1.03
8h	12204.64	849.64	1068.54	164.71	-11.42	10794.12	439.12	-1.13
**IP-10**								
0.5h	404.5	40.5	370.42	130.59	-5.53	470.52	135.3	0.86
2h	2804.86	536.86	1132.22	23.265	-2.48	517.98	155.84	-5.41
4h	17210.31	7366.69	9163.38	151.74	-1.88	7597.30	930.60	-2.26
6h	19789.32	1038.67	55420.98	4500	-0.35	9672.53	437.46	-2.04
8h	16565.13	6156.87	10192.49	489.34	-1.62	11172.54	432.39	-1.48
**MCP-1**								
0.5h	614.62	205.67	355.70	108.91	-5.64	347.31	30.28	-1.76
2h	30473.66	18436.33	14633.51	4079.68	-7.469	8399.78	5757.01	-3.62
4h	72428.12	23993.88	33775.11	3136.75	-23.09	39862.24	4690.32	-1.817
6h	64037.02	32384.97	36905.99	7769.24	-8.24	39414.24	5140.32	-1.63
8h	69453.48	26968.51	44032.97	8741.37	-7.94	42713.20	4070.09	-1.63

* Was calculated by dividing the mean concentration of each cytokine/chemokine induced by *E. coli* by the mean concentration induced by the Fh15-treatment at every time point, the result is expressed as negative result.

**Table 4S.** Number of cells in peripheral blood from rhesus macaques treated with Fh15 alone with and without infection with live *E. coli*. Each experimental group comprised three (n=3) rhesus macaques								
	**E. coli**		**Fh15**		**Fh15-*E. coli***		**Fh15-Fh15-*E. coli***	
**Time (hours)**	**Average No. of cells**	**± SD**	**Average No. of cells**	**± SD**	**Average No. of cells**	**± SD**	**Average No. of cells**	**± SD**
**PBMC**								
0	55634	23191	53256.3	4599.06	50663.0	1165.1	79513.6	12032.7
0.5	3766.5	79.5	36078.0	9465.7	8872.4	12434.2	61523.6	29280.5
2	2150.5	49.5	58620.3	16956.3	15685.9	21887.9	39951.0	29838.4
4	908	14	40514.0	41880.9	83821.0	29154.5	8329.3	4110.1
6	790	94	88505.0	32259.7	86605.6	33359.2	5285.0	2923.5
8	1500.5	69.5	63645.0	30409.1	73734.0	31958.6	64409.6	73869.7
**CMO**								
0	16744	3943	14576.3	3111.6	18261.6	8653.1	32592.6	5703.4
0.5	7	3	9753.6	3372.4	8574.0	1625.6	19023.3	145.22.8
2	68	59	21972.0	15505.4	22177.3	10542.8	7545.0	5504.6
4	35	28	44550.6	23209.7	45525.0	2145.1	594.3	483.9
6	18.5	0.5	51269.6	32871	55844.3	24580.8	348.0	408.1
8	50.5	42.5	32694.3	27591.7	42337.0	22534.5	45783.0	6158.4
**NCMO**								
0	3359.5	1968.5	5843.6	1250.1	3803.6	1609.3	4699.3	12425.3
0.5	26.50	5.5	2757.6	1015.2	1996.3	583.7	18535.3	12185.7
2	80	53	5331.3	2986.1	2801.0	1273.0	1120.6	464.5
4	409	43	6556.0	1151.5	3880.6	1219.6	384.0	322.5
6	426.5	141.5	8316.6	1513.7	6259.3	788.1	144.3	122.3
8	149	120	5521.0	608.1	4181.3	243.5	6272.3	3663.5
**IMO**								
0	3128	1524	1825.3	645.5	2090.3	1001.6	3415.3	1014.3
0.5	22.5	3.5	1795.6	1623.6	740.3	300.3	2990.6	3502.0
2	246.5	123.5	1875.6	1566.1	891.0	218.8	190.6	210.0
4	70.5	6.5	1141.3	514.8	764.6	44.9	15.6	0.94
6	50.5	31.5	2156.6	1258.7	1577.6	488.2	43.3	11.5
8	292	138	1608.3	979.9	1593.3	612.1	1898.3	2187.7
**DCs**								
0	5108	890	362555	39757.8	391353.0	184852.8	2912.3	2072.6
0.5	101.5	92.5	240101.6	45705.4	190228.3	45793.3	3849.6	1860.6
2	58	43	403347.6	78445.6	285749.6	48467.2	2640.0	1348.1
4	13	5	775661.6	50349.0	723955.0	31556.4	874.6	688.7
6	1	0.5	770921.0	53216.2	675481.6	118340.4	104.6	64.5
8	22.5	0.5	700466.3	96590.7	493838.6	187077.3	286.3	272.2
**pDCs**								
0	1620	704	1272.3	808.6	1162.6	972.2	2382.6	989.7
0.5	243	19	1108.0	702.6	986.3	504.8	2049.6	803.1
2	119.5	106.5	1132.6	644.2	988.6	590.3	1101.3	879.1
4	136	134	984.3	851.0	871.66	827.6	83.3	47.6
6	51	41	505.6	308.5	502.6	340.8	107.3	83.8
8	150.5	119.5	144.3	86.4	354.6	211.6	1359.3	908.5
**NKC**								
0	1248.5	20.5	6145.0	1352.8	5552.6	1097.5	9255.0	3204.2
0.5	97	69	3457.0	1299.9	3481.6	1433.2	17061.3	15086.2
2	23.5	6.5	4917.3	2386.2	4605.0	1731.5	1124.0	387.2
4	31	1	9271.6	1869.1	8079.3	1242.9	395.3	334.0
6	41.5	1.5	10087.3	4070.0	9274.0	3623.7	387.6	205.0

8	73	5	4788.0	2061.2	5982.6	3150.6	6852.0	6541.0
**Neut.**								
0	61119	4793	190.6	91.9	447.6	154.4	9625.0	690.0
0.5	38117	10952	308.6	208.0	483.0	211.9	10967.0	8068.0
2	28911	9318	409	244.7	468.3	156.6	22475.0	5780.0
4	17309	100	1046.3	1242.6	345.0	52.7	11778.3	6030.0
6	10158.5	9978.5	255.3	306.6	414.0	310.4	9003.0	4263.1
8	20259.5	5425.5	168.3	82.56	310.0	121.0	24464.6	15608.0

PBMC: peripheral blood mononuclear cells, **CMO**: Classical Monocytes, **NCMO**: Non-classical monocytes, **IMO**: Intermediate monocytes, **DC:** Dendritic cells, **pDC:** Plasmacytoid Dendritic cells, **NKC**: Natural Killer Cells, **Neut:** Neutrophil

References Associated to the Supplementary Information (Table 1S)

1. Ahmed R, Omidian Z, Giwa A, Cornwell B, Majety N, Bell DR, Lee S, Zhang H, Michels A, Desiderio S, Sadegh-Nasseri S, Rabb H, Gritsch S, Suva ML, Cahan P, Zhou R, Jie C, Donner T, Hamad ARA. 2019. A Public BCR Present in a Unique Dual-Receptor-Expressing Lymphocyte from Type 1 Diabetes Patients Encodes a Potent T Cell Autoantigen. Cell 177:1583-1599 e16.
2. Wakeley ME, Shubin NJ, Monaghan SF, Gray CC, Ayala A, Heffernan DS. 2020. Herpes Virus Entry Mediator (HVEM): A Novel Potential Mediator of Trauma-Induced Immunosuppression. J Surg Res 245:610-618.
3. Matzen SMH, Raaschou-Jensen KK, Kallenbach K. 2018. Implementation of the Ogata flow cytometric scoring system in routine diagnostics of myelodysplastic syndrome. Health Sci Rep 1:e90.
4. Fournier N, Jacque E, Fontayne A, Derache D, Dupont G, Verhaeghe L, Baptista L, Dehenne A, Dezetter AS, Terrier A, Longue A, Pochet-Beghin V, Beghin C, Chtourou S, de Romeuf C. 2018. Improved in vitro and in vivo activity against CD303-expressing targets of the chimeric 122A2 antibody selected for specific glycosylation pattern. MAbs 10:651-663.
5. Walk J, de Bree LCJ, Graumans W, Stoter R, van Gemert GJ, van de Vegte-Bolmer M, Teelen K, Hermsen CC, Arts RJW, Behet MC, Keramati F, Moorlag S, Yang ASP, van Crevel R, Aaby P, de Mast Q, van der Ven A, Stabell Benn C, Netea MG, Sauerwein RW. 2019. Outcomes of controlled human malaria infection after BCG vaccination. Nat Commun 10:874.

6. Stroncek DF, Skubitz KM, Plachta LB, Shankar RA, Clay ME, Herman J, Fleit HB, McCullough J. 1991. Alloimmune neonatal neutropenia due to an antibody to the neutrophil Fc-gamma receptor III with maternal deficiency of CD16 antigen. Blood 77:1572-80.

7. Magri G, Comerma L, Pybus M, Sintes J, Llige D, Segura-Garzon D, Bascones S, Yeste A, Grasset EK, Gutzeit C, Uzzan M, Ramanujam M, van Zelm MC, Albero-Gonzalez R, Vazquez I, Iglesias M, Serrano S, Marquez L, Mercade E, Mehandru S, Cerutti A. 2017. Human Secretory IgM Emerges from Plasma Cells Clonally Related to Gut Memory B Cells and Targets Highly Diverse Commensals. Immunity 47:118-134 e8.

8. Carter DL, Shieh TM, Blosser RL, Chadwick KR, Margolick JB, Hildreth JE, Clements JE, Zink MC. 1999. CD56 identifies monocytes and not natural killer cells in rhesus macaques. Cytometry 37:41-50.

9. Edwards JA, Durant BM, Jones DB, Evans PR, Smith JL. 1986. Differential expression of HLA class II antigens in fetal human spleen: relationship of HLA-DP, DQ, and DR to immunoglobulin expression. J Immunol 137:490-7.

10. Halim TYF, Rana BMJ, Walker JA, Kerscher B, Knolle MD, Jolin HE, Serrao EM, Haim-Vilmovsky L, Teichmann SA, Rodewald HR, Botto M, Vyse TJ, Fallon PG, Li Z, Withers DR, McKenzie ANJ. 2018. Tissue-Restricted Adaptive Type 2 Immunity Is Orchestrated by Expression of the Costimulatory Molecule OX40L on Group 2 Innate Lymphoid Cells. Immunity 48:1195-1207 e6.

11. Rueda CM, Presicce P, Jackson CM, Miller LA, Kallapur SG, Jobe AH, Chougnet CA. 2016. Lipopolysaccharide-Induced Chorioamnionitis Promotes IL-1-Dependent

Inflammatory FOXP3+ CD4+ T Cells in the Fetal Rhesus Macaque. J Immunol 196:3706-15.
